# Knowledge and Attitude of Inflammatory Bowel Disease Patients Toward Colorectal Cancer Risk, Its Management, and the Role of Healthcare Providers: A Cross-Sectional Study in the UK

**DOI:** 10.1093/crocol/otad067

**Published:** 2023-10-24

**Authors:** Fiza Khan, Christine Norton, Wladyslawa Czuber-Dochan

**Affiliations:** Florence Nightingale Faculty of Nursing, Midwifery and Palliative Care, King’s College London, London, UK; Florence Nightingale Faculty of Nursing, Midwifery and Palliative Care, King’s College London, London, UK; Florence Nightingale Faculty of Nursing, Midwifery and Palliative Care, King’s College London, London, UK

**Keywords:** inflammatory bowel disease, colorectal cancer, cancer awareness

## Abstract

**Background:**

Inflammatory bowel disease (IBD) increases the risk for colorectal cancer (CRC). Limited literature exists on patients’ knowledge of CRC risk and management. Attitude toward doctor-recommended management and the role of healthcare providers (HCPs) in CRC risk awareness remain unexplored. This study aimed to fill the gap in knowledge about CRC risk awareness and management in IBD patients in the UK.

**Methods:**

This cross-sectional internet-based study was conducted in April–July 2019. Adult (>18 years) IBD patients with a confirmed diagnosis for 2 years and adequate command of English language were invited from non-Natinal Health Services sources. A self-designed and piloted questionnaire with open- and closed-ended questions was used. Closed-ended data were analyzed using descriptive statistics and open-ended responses were analyzed using content analysis.

**Results:**

Ninety-two participants (52.5% Crohn’s disease and 67.5% females) responded. Around 88% knew that IBD increased CRC risk. Only 20.7% were aware of colonoscopy as the best screening tool; 88% were unaware of screening initiation time. Almost 90% would agree to a doctor’s recommendation of colonoscopy. For dysplasia with 10% risk of CRC, 46.7% would not agree with colectomy. Some 48% reported to have never had a discussion about the risk of CRC in IBD with their HCPs, while 58% were not informed of the role of screening and surveillance in managing CRC risk.

**Conclusions:**

IBD patients were poorly aware of CRC risk management and had mixed willingness to comply with a doctor’s recommendation. HCP’s role in cancer knowledge dissemination was suboptimal and patients desired more information.

## Graphical Abstract

**Graphical Abstract:**
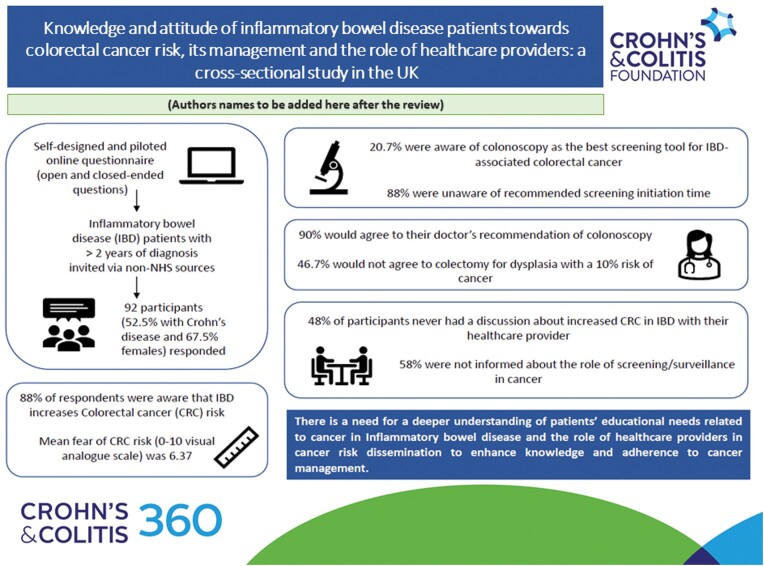

**Key Message**

- Inflammatory bowel disease (IBD) patients have an increased risk of colorectal cancer (CRC) and their knowledge of this risk, its management, and the role played by their healthcare providers in raising cancer risk awareness remains unknown in the UK.
- We found that patients were well informed of the increased cancer risk, were poorly informed about screening timeline, had a mixed attitude toward recommended treatment, and the role of healthcare providers in cancer risk education was suboptimal.
- This study highlights the cancer risk-related educational needs in IBD patients to improve adherence and acceptance of recommended management.

## Introduction

Inflammatory bowel disease (IBD) is a chronic remitting and relapsing inflammatory condition affecting the gut. Ulcerative colitis (UC) and Crohn’s disease (CD) are the most common subtypes of IBD.^1,2^ In the UK, approximately 146 000 people have UC and 115 000 people have CD.^3,4^

Chronic inflammation in IBD increases the risk for dysplasia and subsequent colorectal cancer (CRC) and is responsible for 10%–15% of deaths in IBD patients.^5,6^ A meta-analysis showed a cumulative risk of CRC in UC of 1.6 % at 10 years, 8.3 % at 20 years, and 18.4 % at 30 years after diagnosis.^7^ A similar risk of CRC has also been reported in CD.^8^ The risk increases with increasing disease duration, disease severity and chronicity, positive family history of CRC, disease onset before 20 years of age, and coexisting primary sclerosing cholangitis (PSC).^7,9,10^

To ensure timely detection of dysplastic changes, the 2023 European Crohn’s and Colitis Organization (ECCO) consensus recommends screening colonoscopy to be carried out 8 years after the first symptom onset followed by a surveillance colonoscopy every 2–3 years.^11^ Detection of low-grade flat dysplasia is associated with a 20% chance of having cancer and 50% chance of progression to high-grade dysplasia within 5 years. Therefore, colectomy is recommended as a definitive treatment for dysplasia and cancer prevention.^12–14^

It has been shown that higher knowledge of disease enhances patient adherence to treatment and also reduces treatment costs.^15–17^ Many studies have assessed IBD patients’ disease-related knowledge using the Crohn’s & Colitis Knowledge Questionnaire (CCKNOW) which includes a single question about CRC risk.^18^ However, literature on IBD patients’ knowledge and awareness of CRC risk, their fear of this complication, and their knowledge of its management is sparse.^19–23^ Although authoritative guidelines and recommendations for managing CRC risk in IBD exist, patients’ attitude and willingness to adhere to these recommendations are less explored.^22,24^ The role of physicians as the most reliable source of information has been established in studies on other diseases.^25^ It is essential that patients’ perception of their healthcare provider’s (HCP) role be assessed to see if they are satisfied with how they are educated about their cancer risk or if they would prefer being informed and managed differently.

It is important to assess the current understanding of IBD patients about their cancer risk and its management, to identify if there is a knowledge gap. Thereafter, appropriate educational interventions can be designed and applied with the aim of enhancing knowledge and adherence to preventive or diagnostic modalities available for CRC. It is also essential that patients’ attitude toward recommended CRC treatment and factors that motivate agreement to recommendations be assessed, to understand what drives agreement, and how patient adherence to recommended guidelines can be improved. Knowing patients’ perception of HCP role would help notify HCPs of patients’ information needs, helping them modify their clinical practice to meet patients’ expectations and educational demands, creating an environment conducive to informed and shared decision-making. Therefore, this study aimed to assess UK patients with IBD’s knowledge, fear of CRC risk and attitude toward CRC management, and the role of HCPs in CRC risk awareness.

## Materials and Methods

### Study Design and Data Collection

This questionnaire-based cross-sectional study was conducted from April to July 2019. Potential participants were approached via invitation emails sent to a database of people with IBD who had participated in past research studies with our group and who had previously consented to be approached for future studies and to the email mailing list of Bowel and Cancer Research Charity UK. The study was also advertised on the IBD Relief website and the Bowel and Cancer Research Charity Facebook page.

The invitation email included a participant advertisement and participant information sheet (PIS) and a link to the online survey which incorporated online consent. Participants had the option to use the link directly or send their postal address for a paper questionnaire. Only 1 participant opted for the latter and was sent a consent form, PIS, and paper questionnaire with a stamped and addressed envelope for the return of the questionnaire.

### Sample Size and Study Participants

This study was a descriptive exploratory study. The aim was to recruit 100 participants, informed by the sample size of a previously published similar questionnaire-based study.^22^ Adult participants (18 years and above) with self-confirmed diagnosis of IBD for at least 2 years and adequate command of written English to understand the study questionnaire and give consent, were included in the study.

### Data Collection Instrument

Data were collected using a piloted questionnaire which was designed following a detailed literature review. The final questionnaire included 38 questions (5 sections) and adapted the 12-item questionnaire on CRC risk knowledge and fear by Lopez et al.^22^ Both open- and closed-ended questions were included that assessed the following study outcomes: Knowledge of IBD complications and fear of CRC (closed questions and visual analog scales); knowledge of CRC risk (closed questions); knowledge of CRC management (closed questions); patient attitude toward a doctor’s recommendation of CRC risk management (closed- and open-ended responses); the role of HCPs in educating IBD patients about CRC risk and management (closed- and open-ended responses; see Supplementary Data, Content 1).

### Data Analysis

Quantitative data were analyzed using Statistical Package for the Social Sciences (SPSS) Version 25.^26^ Patient demographic data and closed-ended question responses were analyzed using descriptive statistics and reported as numbers and frequencies for categorical variables and means with standard deviations (SDs) for continuous variables.

Open-ended question responses were analyzed using content analysis. The qualitative responses were read thoroughly to examine the data and become familiar with the responses, followed by generating codes for repeating concepts. Descriptive coding was employed to code the responses and tally marks were used to determine the frequency of each code. The final codes with their respective frequencies are reported.

## Results

### Participant Characteristics

Ninety-two responses were received (91 online and 1 paper). The response rate cannot be calculated because the study was advertised via social media. More than half of the respondents had CD (52.2%) and two-thirds (67.5%) were female. The mean participant age was 53.23 years (SD ± 15.8) and the mean disease duration was 20 years (SD ± 13.52). Seventy-four percent were under the care of a gastroenterologist, while 21.7% followed up with an IBD nurse. Most participants were White British (82.6%) and 54.3% had an education level of university degree and above (Table 1).

**Table 1.** Participant characteristics.

Demographics and clinical characteristics	Number of participants reporting	Means/percentages
IBD type	92	
UC	42	45.7%
CD	48	52.2%
IBD unclassified	2	2.2 %
Sex		
Female	62	67.5%
Male	30	32.7%
Age	90	53.23 (SD 15.8)
Length-of-time since IBD diagnosis (years)	90	20.14 (SD 13.52)
Follow-up/care provided for IBD by	92	
Gastroenterologist	68	73.9%
IBD specialist nurse	20	21.7%
General practitioner	4	4.3%
Education level	92	
No formal qualification	4	4.3%
Vocational qualifications (NVQ)	6	6.5%
School-level qualifications (GCSE, O Level, CSE)	20	21.7%
Advanced school-level qualifications (A Levels, Scottish Highers)	12	13%
University degree and above (BSc, BA, MSc, PhD)	50	54.3%
Ethnicity	92	
White—British	76	82.6%
White—Irish	6	6.5%
White—any other White background	3	3.3%
Asian or Asian British—Indian	3	3.3%
Asian or Asian British—any other Asian background	1	1.1%
Arab	1	1.1%
Mixed—White and Black Caribbean	1	1.1%
Any other ethnic origin group	1	1.1%

Abbreviations: CD, Crohn’s disease; IBD, inflammatory bowel disease; SD, standard deviation; UC, ulcerative colitis.

### Knowledge of IBD Complications and Fear of CRC

Participants were asked about their knowledge of the most common complications of IBD followed by the complication they feared the most. More than two-thirds (77.3%) reported increased risk of bowel cancer, followed by bowel obstruction (25%), bowel perforation (25%), and intestinal fistulas (25%) as the most common complications of IBD. The need for surgery, particularly total or subtotal colectomy, was also mentioned by one-fifth of the respondents (21.6%).

**Figure 1.**
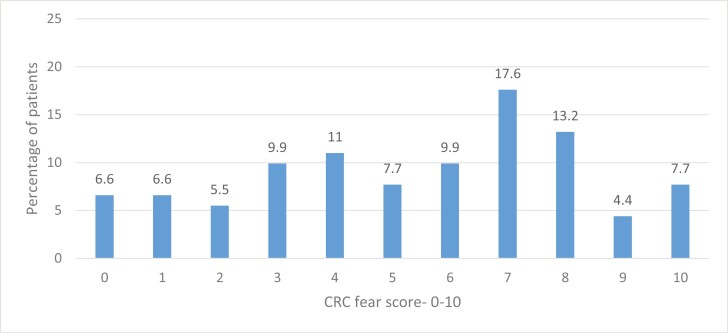
Visual Analog scale—fear of colorectal cancer (CRC).

The most feared IBD complication reported was risk of CRC (63%) followed by bowel obstruction (7.6%), bowel perforation (5.4%), and need for surgery (4.3%). The mean CRC fear on the visual analog scale was 6.37 (SD ± 2.8; median 7) with more than half of the respondents scoring their fear above 6 on a scale of 0–10 (Figure 1).

### Knowledge of CRC Risk in IBD

Eighty-eight percent of the participants were aware of the increased risk of CRC in IBD, 3% believed that IBD does not increase cancer risk, while 9% were unsure. The main sources of knowledge were gastroenterologists (46.7 %) and the internet (44.6%), followed by newspapers (8.7%), charity organizations (8.7%), other patients (7.6%), and general practitioner (GP; family doctor; 6.5%).

Two-thirds of participants were aware of active inflammation (76%), bowel polyps (76%), family history of cancer (79%), and increased extent (64%) and duration of IBD (64%) as risk factors for CRC. Only one-fifth (20.7%) were familiar with the role of IBD diagnosis before the age of 20 years in increasing CRC risk, while the majority (83.7%) were unsure of coexisting PSC as a risk factor for CRC in IBD.

### Knowledge of CRC Risk Management in IBD

**Figure 2.**
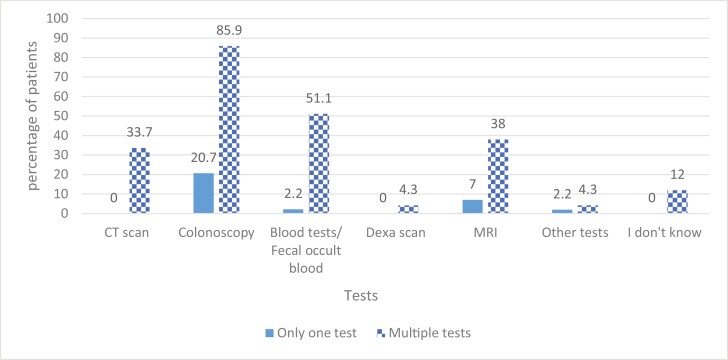
Knowledge of tests used for colorectal cancer (CRC) screening.

Most respondents thought that multiple tests were used for CRC screening. The most common test suggested was colonoscopy (85.9%) followed by blood tests and fecal occult blood test (51.1%). Only 20.7% of respondents correctly identified colonoscopy as the only test used to screen for bowel cancer, while 12% of participants did not know which test was used (Figure 2).

**Figure 3.**
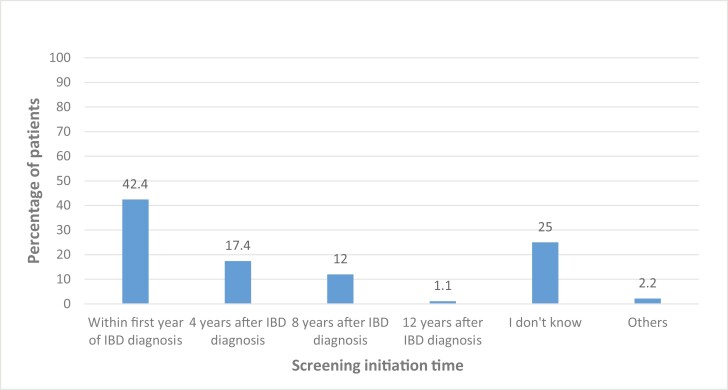
Knowledge of colorectal cancer (CRC) screening initiation time.

Forty-two percent of the participants believed that screening for CRC should be initiated within the first year of IBD diagnosis, while around 17% thought it is to be initiated within 4 years after IBD diagnosis. Some 12% thought it started 8 years post-diagnosis (Figure 3), and 25% of participants were not sure about the screening initiation time for CRC in IBD.

### Patient Attitude Toward Doctor’s Recommendation of Colonoscopy for CRC Risk

Ninety percent of the respondents said that they would agree with doctor’s recommendation for screening, 1% of participants said they would refuse, while 9% were unsure. The most common reason, as stated by the participants, for agreeing to colonoscopy was that it helps catch cancer at an early stage and ensures early treatment to improve prognosis and survival (*n* = 28). This was followed by trusting the doctor’s recommendation (*n* = 13), undergoing screening would help gain reassurance of good health (*n* = 7), and having a positive family history of cancer that encourages participants to stay careful (*n* = 4).

The only participant who disagreed with the doctor’s recommendation felt it was unnecessary to undergo the procedure as they had already been diagnosed with CRC. Other reasons for being unsure about agreeing with the doctor’s recommendation included: Not having enough knowledge about colonoscopy and how frequently it would be needed (*n* = 5), being unsure due to other health problems (*n* = 1), and being only willing to agree if the cancer risk were higher than 10% (*n* = 1).

### Patient Attitude Toward Doctor’s Recommendation of Colectomy With a 10% Risk of CRC

The questionnaire gave a scenario of dysplasia with a 10% risk of progressing to cancer. Regarding willingness to comply with a doctor’s recommendation of colectomy for dysplasia, 32.2% said they would agree, 21.1% would say no, and 46.7% were unsure about their decision.

The most common reasons for agreeing to colectomy were reduction in risk of CRC as well as help in getting rid of IBD symptoms (*n* = 7), 10% risk being high enough to act and not leave the dysplasia untreated (*n* = 5), having a colectomy rather than risking cancer (*n* = 5), and trusting the doctor’s guidance (*n* = 4).

Reasons for disagreeing with doctor’s recommendation included: 10% risk of CRC not being high enough to undergo this drastic surgery (*n* = 13), an interest in exploring other treatment options for dysplasia (*n* = 5), monitoring the lesion regularly instead of colectomy (*n* = 4), and interest in receiving more information (*n* = 3).

Patients who were unsure about agreeing with a doctor’s recommendation of colectomy gave the following reasons: Not having enough information to make a life-changing decision (*n* = 11), an interest in more information about the effectiveness, advantages, disadvantages, and possible complications of colectomy before deciding to proceed (*n* = 10), being fearful of major surgery and the possibility of living with a colostomy (*n* = 6), and the need to understand what the 10% risk of CRC entails (*n* = 5).

### Role of Healthcare Providers in Educating IBD Patients About CRC Risk and Management

Forty-eight percent of respondents reported that they have never discussed the increased risk of CRC in IBD with their HCP. Of those who were informed of the risk, 59% were satisfied and 33% were dissatisfied with the information shared. The most common reason for satisfaction was being informed about CRC risk without provoking anxiety (*n* = 2). Not being provided with adequate detail about the CRC risk (*n* = 7) and only being informed about the risk of cancer stemming from the use of IBD medication but not from IBD itself (*n* = 4), were the 2 most common reasons for dissatisfaction. Patients desired more information about the general percent risk of CRC with IBD as well as individualized risk and factors that increase the risk (*n* = 17), information on preventive measures and how to decrease the risk (*n* = 6), as well as signs and symptoms to watch out for in case of CRC (*n* = 6).

Fifty-eight percent of participants suggested that they were not informed by their HCP of the role of screening and surveillance in managing CRC risk. Of those who were informed, 68.4% were satisfied with the information provided; however, reasons for satisfaction were not provided. The most common reason for dissatisfaction was that the role of screening and surveillance were very briefly discussed or discussed only when prompted (*n* = 5). Of the 58% who were not informed about the role of screening and surveillance in IBD, 38 patients wanted more information. Participants wanted to know what screening and surveillance involve, what it aims to achieve, the tests used for screening, and the frequency of screening tests used (*n* = 18). They were also curious about their personal need for screening and wanted to know the advantages of screening and an early cancer diagnosis (*N* = 9).

### Patients’ Suggestions for Healthcare Providers for Managing CRC Risk in IBD

Thirty-six respondents gave suggestions for HCPs. Overall, respondents wanted HCP to provide more information, some specific to cancer and services available for cancer and others more about preventative aspects of cancer (*n* = 7). While some respondents wanted their HCP to be upfront and thorough while communicating cancer risk (*n* = 5), some would only like to be informed if, and when it was necessary, to save themselves from stress (*n* = 6).

## Discussion

This study found that increased risk of CRC was the most feared IBD complication for 63% of respondents, corresponding to a previous study.^27^ However, 2 studies have shown lower fear levels.^22,28^ The fear of CRC on the visual analog scale was also higher than seen in previous studies (6.37 vs. 5.42 and 4.79, respectively).^22,29^ Moser et al. found that the most common concerns of IBD patients were having an ostomy bag, side effects of IBD medication, and surgery.^30^ This contrasts with the current study findings where less than 5% of participants feared surgery and no fear of the effect of IBD medications was reported. The likely explanation for the varying level of fear could be the difference in study populations or cross-cultural variations in perception of disease-related fear among IBD patients.^31^ Additionally, participants opted into the current study and knew that the focus was on CRC, so may have been self-selected as fearful of this. However, it is evident that CRC risk is one of the most concerning complications for IBD patients and therefore, requires urgent attention for patient education regarding the extent of risk and how it can be managed, to avoid unwarranted stress.

Around 88% of participants knew that IBD increases CRC risk. A similar high awareness level has been reported in 2 studies in UC patients,^19,24^ while lower awareness was seen in 2 other studies.^21,22^ Around two-thirds of the participants correctly identified most of the risk factors for CRC in IBD. However, poor awareness of coexisting PSC and disease diagnosis at a younger age (<20 years) as risk factors was found, corresponding to the findings of Popa et al.^23^ These variations in awareness may be attributed to the differences in participant characteristics, awareness strategies and healthcare systems of different countries and methods of data collection employed by the studies. Studies reporting higher CRC risk awareness included IBD patients with longer disease duration and higher education level.^19,24^ Most of the current study participants had a disease duration of over 20 years and a university education, factors that could explain the higher risk awareness.

The main sources of CRC risk knowledge for the current study participants were gastroenterologists and the internet. Doctors, particularly gastroenterologists, have been regarded as the preferred source of information for IBD patients in several studies.^22,29^ The use of the internet to attain disease-related information is also very common.^22,32^ However, the reliability of information available on the internet cannot be ascertained as many websites give inaccurate information, which can potentially lead to patient misinformation.^33,34^ Therefore, it is essential that clinicians guide patients about reliable online resources available to avoid misinformation.

Even though 86% of participants identified the use of colonoscopy in screening for CRC, only one-fifth identified it as the only test used for screening and surveillance. A similar high awareness of screening tests was seen in 2 European studies.^22,23^ Almost half of the participants also mentioned that fecal occult blood testing (FOBt) can be used for CRC screening. The UK Bowel Cancer Screening Program uses FOBt to screen for CRC in the general population.^35,36^ Although FOBt does not have a role in IBD-associated CRC screening, the high publicity and awareness of general bowel cancer screening program may have led to the patient misinterpretation of the role of FOBt in screening for IBD-associated CRC as well.

According to the 2023 ECCO guideline, screening should be initiated at 8 years post first symptom onset.^11^ Only 12% of respondents in this study suggested that CRC screening should be initiated at 8 years post-IBD diagnosis. This low awareness is concerning as most of the participants in this study had a disease duration of more than ten years, so should have already been screened and enrolled in a surveillance program for bowel cancer. Similar awareness of screening initiation has been reported for Irish IBD patients, while higher knowledge of screening initiation is reported in 2 European studies.^22,23^ It is known that gastroenterologists have a varied perception of cancer risk with dysplasia and practice screening in a disordered manner, factors that may affect how patients perceive the screening frequency and may also explain the current study findings.^37–39^

Ninety percent said that they would agree to doctor’s recommendation of colonoscopy as it would help improve survival by ensuring early cancer detection and treatment and because they trust their doctor’s guidance. A comparatively lower willingness of UK IBD patients for colonoscopy was reported by Robinson et al., where only 54% of patients would agree to undergo colonoscopy.^19^ This possibly indicates that patients’ perception of screening, their educational needs, and their willingness to undergo screening have improved over the last 20 years. However, both these studies assessed willingness for screening in a hypothetical situation and it can be presumed that patients’ willingness may differ when they are presented with a choice of colonoscopy in clinical practice. Therefore, it is essential that IBD patients are educated about the advantages and disadvantages of screening, to ensure that reported attitude translates into actual practice of improved screening adherence and reduced CRC risk.

Half of the present-study patients were unsure about agreeing to colectomy mainly due to lack of enough information while 21.1% would disagree, as a 10% risk of cancer was not high enough to undergo this drastic surgery. These findings are similar to a French study where 51% of IBD patients would refuse recommendations of colectomy mainly due to fear of an ostomy bag and serious infections.^22^ In contrast, higher willingness was reported in 2 European studies where 50% of patients said they would agree with their doctor’s recommendations of colectomy.^21,23^ These studies assessed the attitude toward a 20% risk of progression to CRC, higher than that used in the current and the French study, which may explain the slightly greater willingness reported. The most significant barrier to decision-making and following a doctor’s recommendation was the lack of sufficient information about colectomy, providing patients with guidance about what colectomy entails. Addressing these concerns may impact patient perception and attitude toward this surgery.

Forty-eight percent of the patients never had a discussion with their doctor or IBD nurse about the increased risk of CRC, while 58% reported that they were never told about the role of screening and surveillance for CRC in IBD. Of those who had a discussion, one-third were unsatisfied as the information shared was insufficient and brief. These findings are concerning, given that majority of the participants in this study had a disease duration of greater than 10 years and were at a higher risk of CRC, hence, were expected to have been informed of the risk. Consequently, HCPs may play a suboptimal role in knowledge dissemination and education regarding CRC risk and its management in IBD patients. No clear guidelines exist to guide HCPs about the appropriate information and time to start discussing cancer in IBD. Therefore, the topic might not be discussed in-depth with the patients to avoid unwarranted stress and anxiety. Another possibility may be that HCPs themselves are not well informed of the CRC risk in IBD, as reported by Kabir et al. who found a wide variation in self-perceived cancer risk with low- and high-grade dysplasia among gastroenterologists.^39^ As no literature exists regarding factors that prevent effective CRC education of IBD patients by the clinicians, the exact reasons for poor cancer risk dissemination remain unknown.

Patients who never had a discussion with their HCPs about CRC risk and its management wanted more information. Emphasis was on the need for information from reliable sources, like the National Health Service, about the general cancer risk as well as individualized risk of CRC in IBD. They suggested that HCPs should discuss the factors that increase risk, the measures that could prevent risk, and signs and symptoms to watch out for. Although most participants wanted the HCPs to be open and upfront with patients while discussing CRC risk, some preferred being informed only, if necessary, to avoid stress. As no previous studies have assessed the information needed for CRC risk in IBD, comparisons can not be drawn. However, it is evident that patients with IBD are keen on learning more about their cancer risk. Like disease management and CRC risk management in IBD, patient education must also be individualized to cater to individual patient needs. The desired information must be conveyed in an effective manner to avoid stress and HCPs must encourage 2-way conversations to enable patients to clear confusions and concerns about their CRC risk and its management. It is also essential that easy access to further educational sources be provided to enable patients to revisit information as desired and avoid less reliable sources with misleading information. These efforts shall lead toward higher awareness which may translate into positive perceptions and increased adherence to the recommended management options for CRC. It may also promote an educational environment conducive to shared decision-making and patient empowerment to improve the mortality of cancer in this high-risk population.

This study had some limitations. A sample size calculation was not done as the study was purely descriptive and adapted from Lopez et al.^.22^ Self-selection is likely to have introduced selection bias and may have attracted IBD patients with higher concerns and more knowledge than most about CRC, preventing the results from being generalizable. As participants were recruited via various sources, extent of dissemination of the questionnaire could not be ascertained and response rate could not be calculated. Some participants did not answer all questions leading to some missing data. The questionnaire posed a hypothetical situation (10% CRC risk), and responses may be different from a real dysplasia diagnosis. Recall bias may have influenced responses about past interactions with HCPs. The questionnaire asked for screening initiation time based on disease duration since IBD disease diagnosis instead of duration since the symptom onset, as suggested by the ECCO guidelines. Therefore, the result may not reflect true patient awareness of screening initiation time and must be interpreted cautiously. As the study questionnaire did not collect data on disease site, extent, and severity, this could influence the answers about complications. Different disease phenotypes can lead to different complications, and this may affect patient awareness and fear of these complications. The questionnaire inquired about only 5 possible complications of IBD, which could lead to bias, as this questionnaire was related to CRC risk. Therefore, the results should be interpreted cautiously. Additionally, the questionnaire did not collect data on disease burden. Disease burden, duration, and who the patients were under the care of (specialist vs. GP) may potentially impact a patient’s awareness level and lead to bias. A subgroup analysis could highlight these important associations in a future larger study. However, this was beyond the scope of this simple descriptive survey. Despite the limitation, this study is the first to primarily explore knowledge and awareness of CRC risk and its management in patients with IBD in the UK. Although the study questionnaire was designed after an extensive literature review and was piloted among IBD patients, it was not validated. Nonetheless, it is the first author-designed and piloted questionnaire covering detailed aspects of CRC risk and management knowledge in IBD, that fits well with the aim of the current study. This questionnaire can be further tested and validated to increase its’ application in future research.

In conclusion, this study is the first to assess IBD patient’s knowledge and fear of CRC risk and their willingness to adhere to HCPs’ recommendations for risk management in the UK. Patients were well informed of the increased CRC risk and the use of colonoscopy for screening but were poorly aware of when screening was initiated. Patients had a positive attitude toward recommendations for colonoscopy. However, mixed willingness toward colectomy for dysplasia was reported, mostly due to lack of knowledge of what the surgery entailed. HCP’s role in cancer knowledge dissemination was suboptimal and patients desired more knowledge. There is a need for patient education to enhance knowledge and adherence to available management options to improve morbidity and mortality in this high-risk population.

## Supplementary Material

otad067_suppl_Supplementary_Material

## Data Availability

Data not publicly available.
