# Supplementary material for: Knowledge and Attitude of Inflammatory Bowel Disease Patients Toward Colorectal Cancer Risk, Its Management, and the Role of Healthcare Providers: A Cross-Sectional Study in the UK
Source: Crohns Colitis 360. 2023 Oct 24;5(4):otad067. doi: 10.1093/crocol/otad067 (PMC10629216; doi:10.1093/crocol/otad067)
Supplement: otad067_suppl_Supplementary_Material [file otad067_suppl_supplementary_material.docx]

**Knowledge and attitude of inflammatory bowel disease patients towards cancer risk, its management, and role of healthcare providers: a cross-sectional study in the UK**

**Supplement**

**Contents**

[**Supplementary Data Content 1.** Participant Information Sheet, Consent form and Study Questionnaire 2](#_Toc103182954)

# **Supplementary Data Content 1.
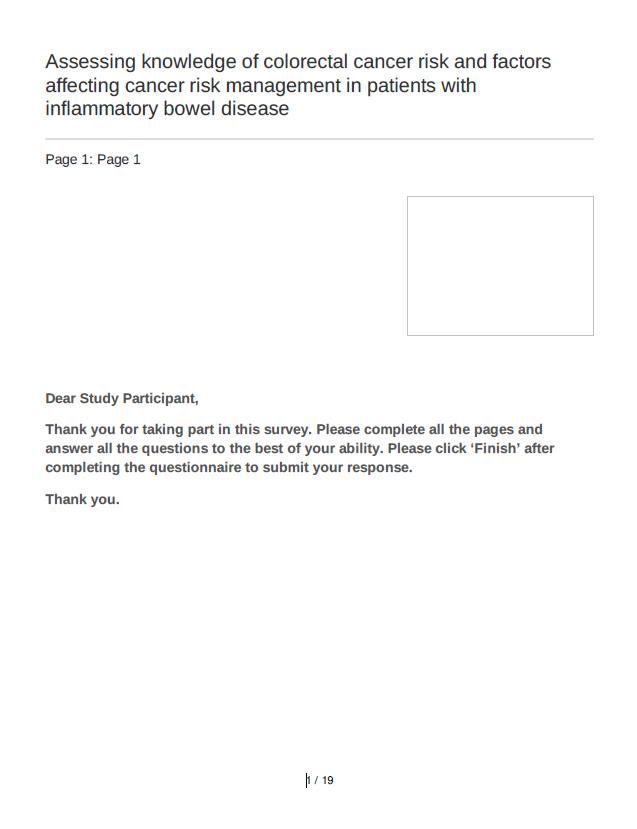
 Participant Information Sheet, Consent form and Study Questionnaire**

**Participant Information Sheet**

**Ethical Application Reference Number: 10417**

**Ethical Clerance Reference Number: HR-18/19-10417**

**
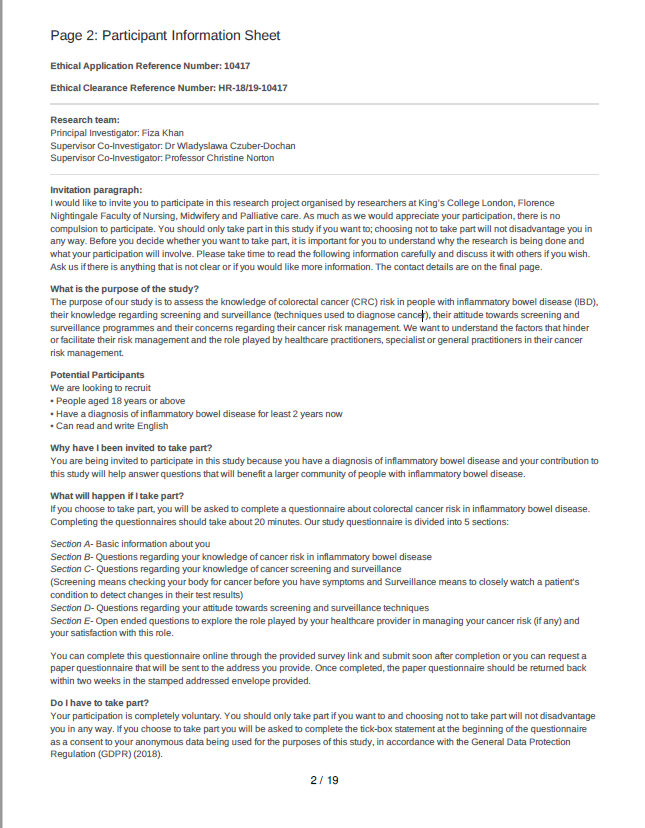
Research Team: (names to be added after the review process)**

**
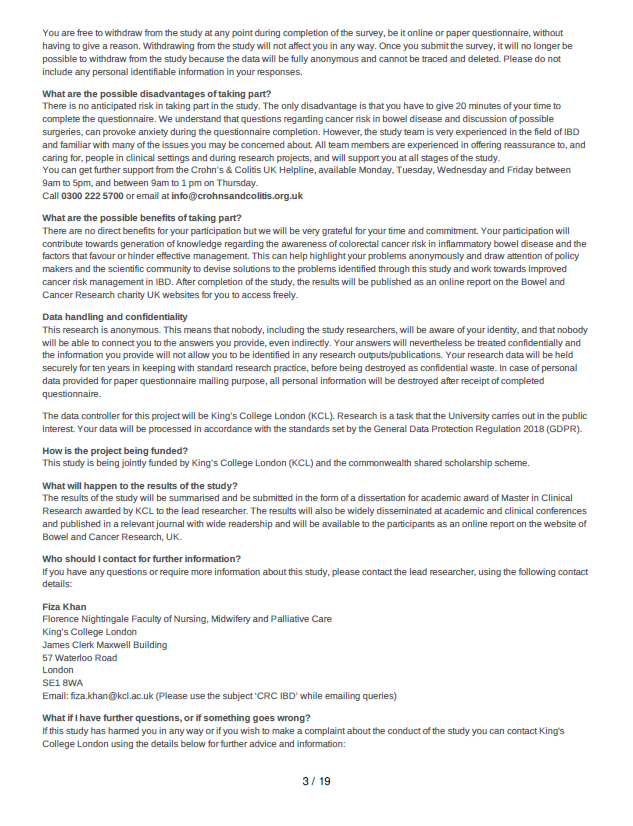
**

**
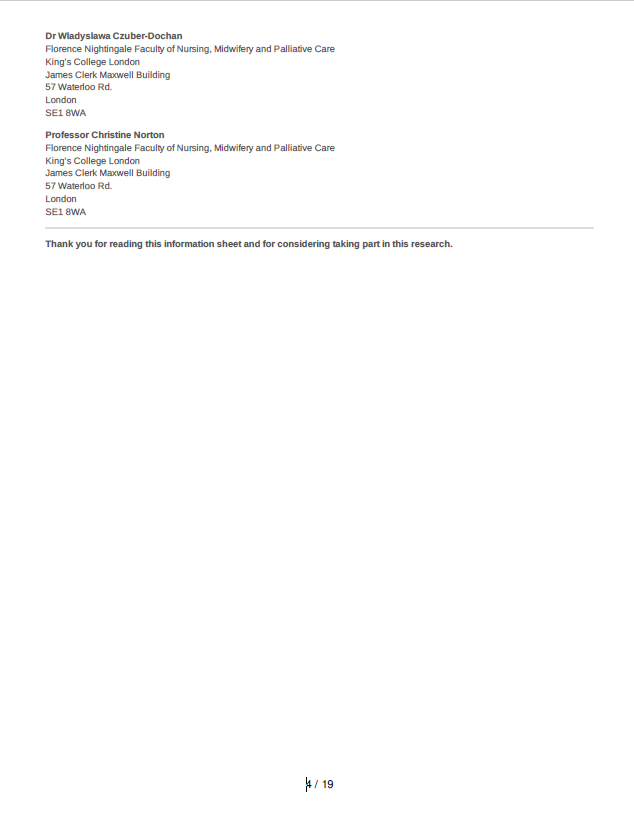
**

**
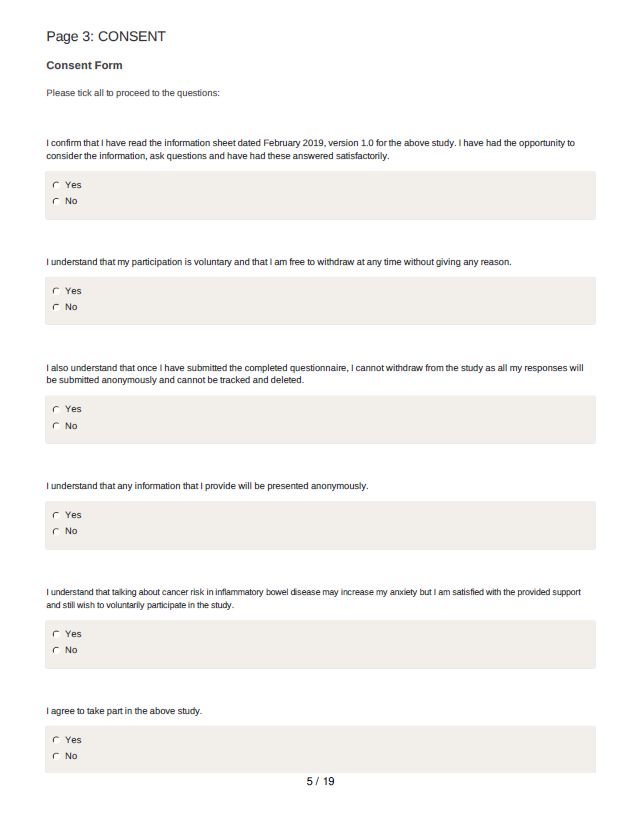
**

**
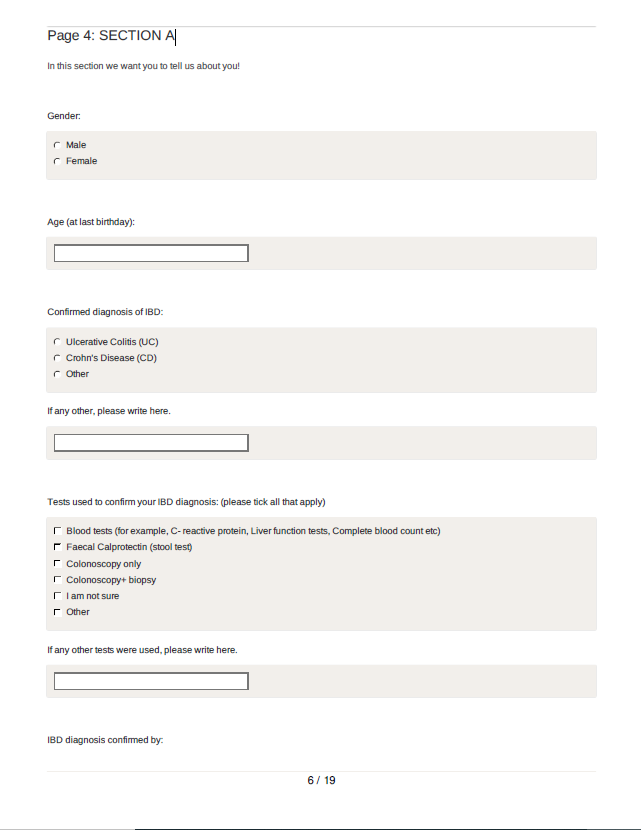
**

**
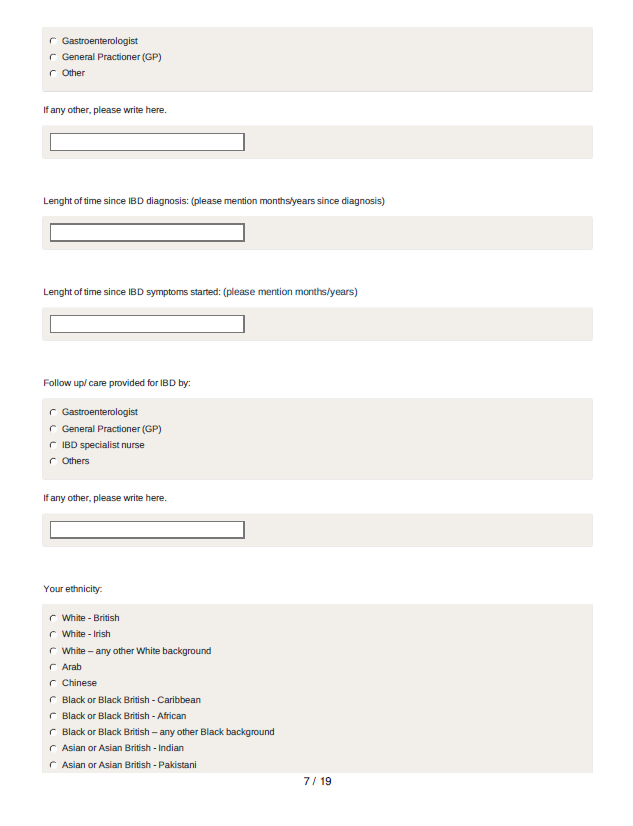
**

**
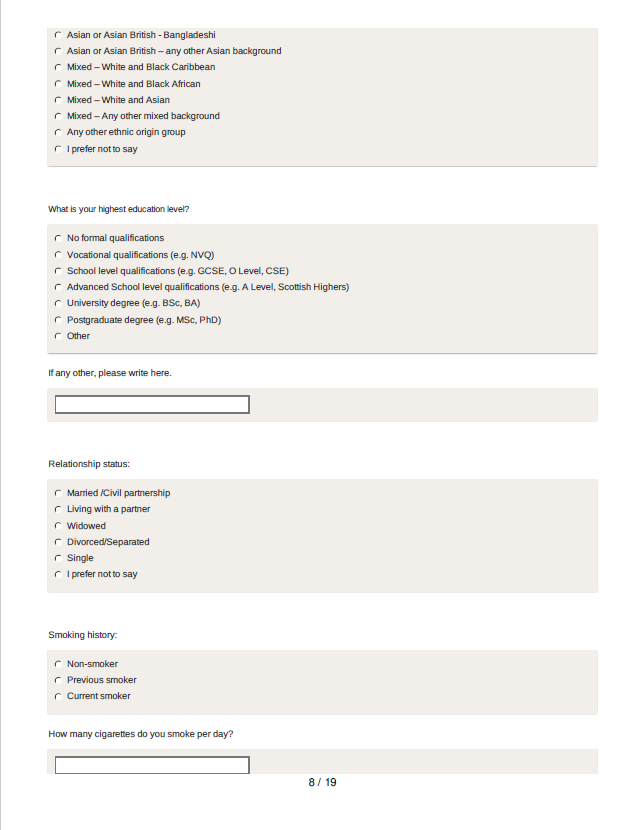
**

**
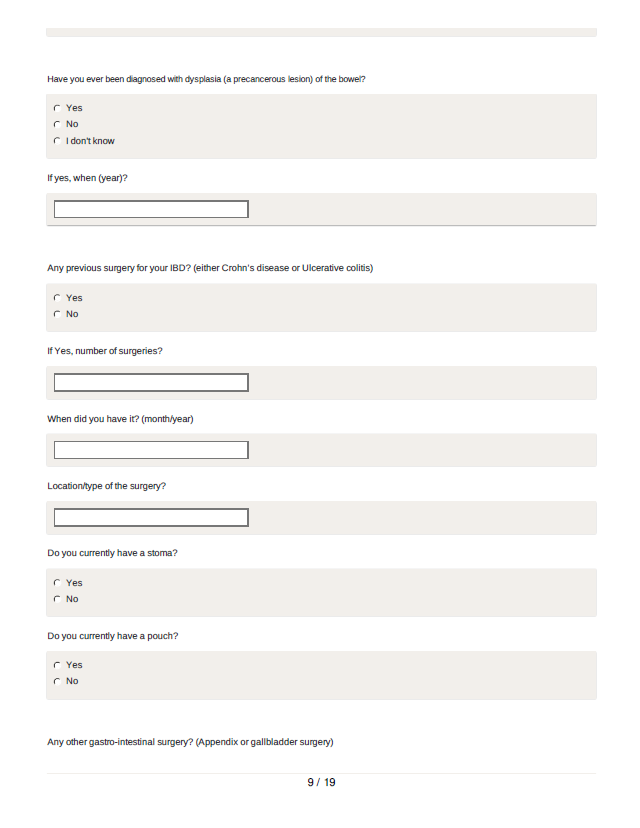
**

**
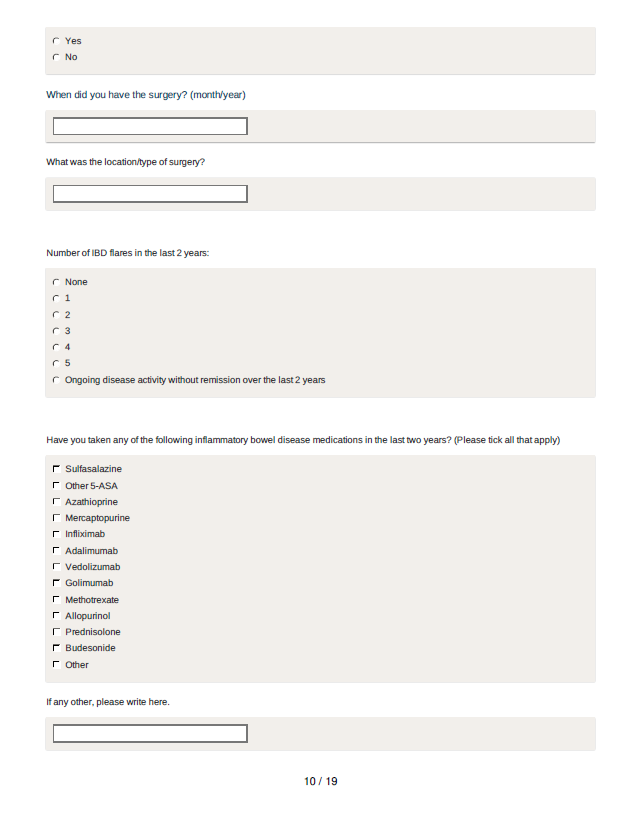
**

**
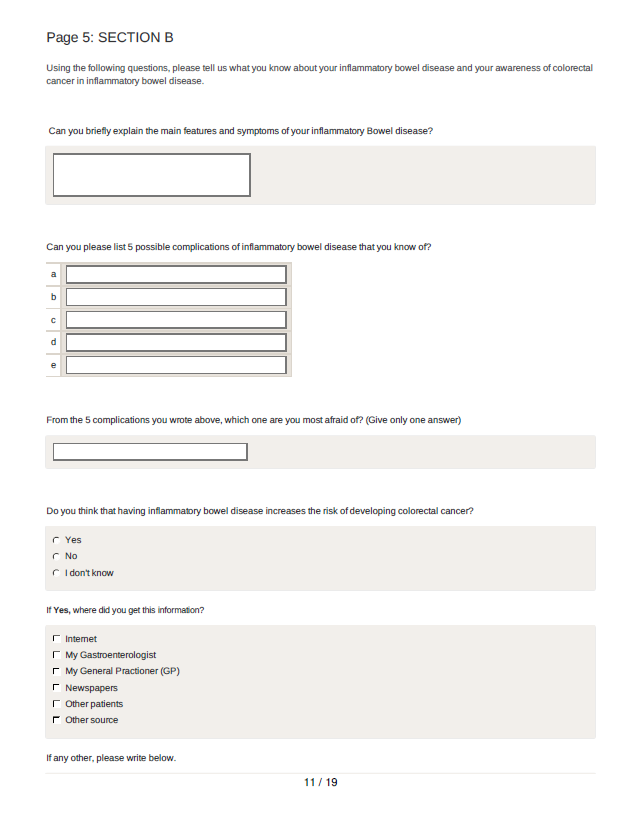
**

**
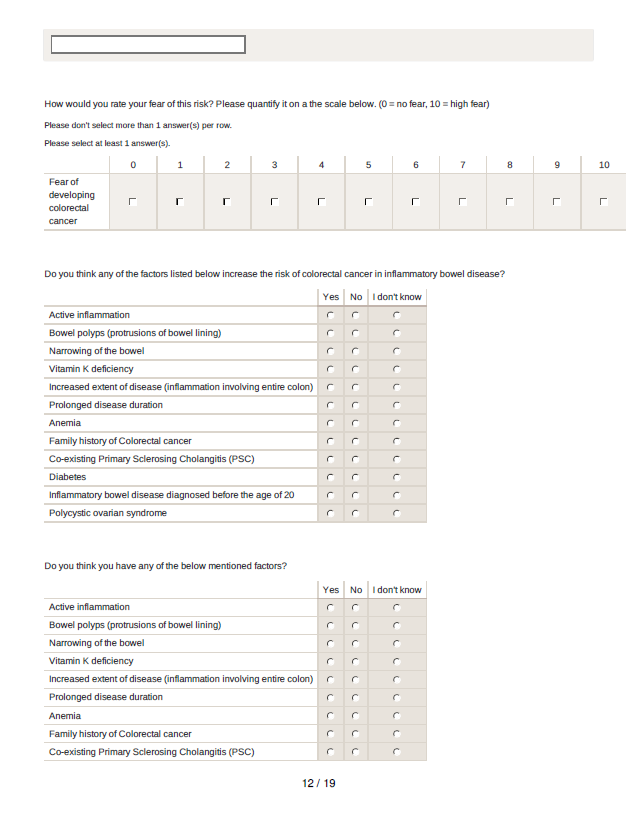
**

**
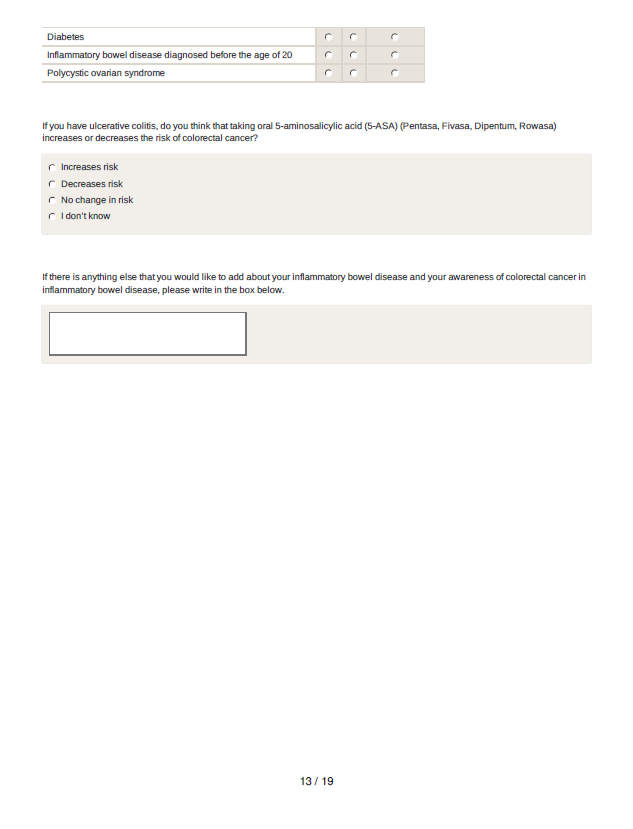
**

**
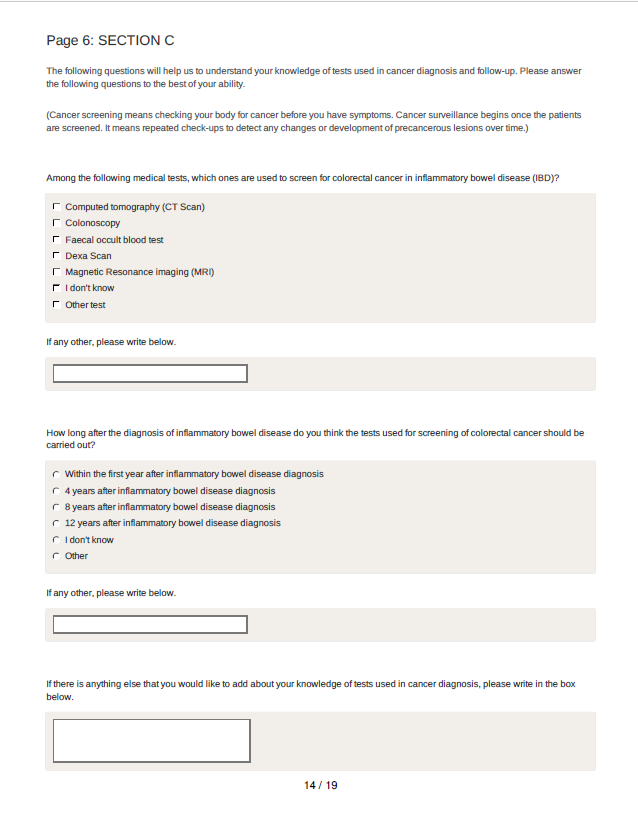
**

**
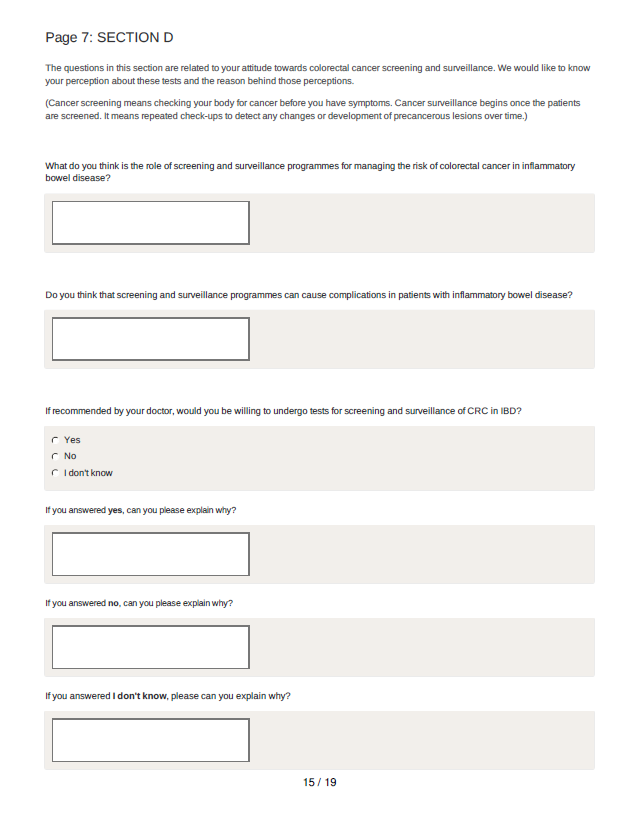
**

**
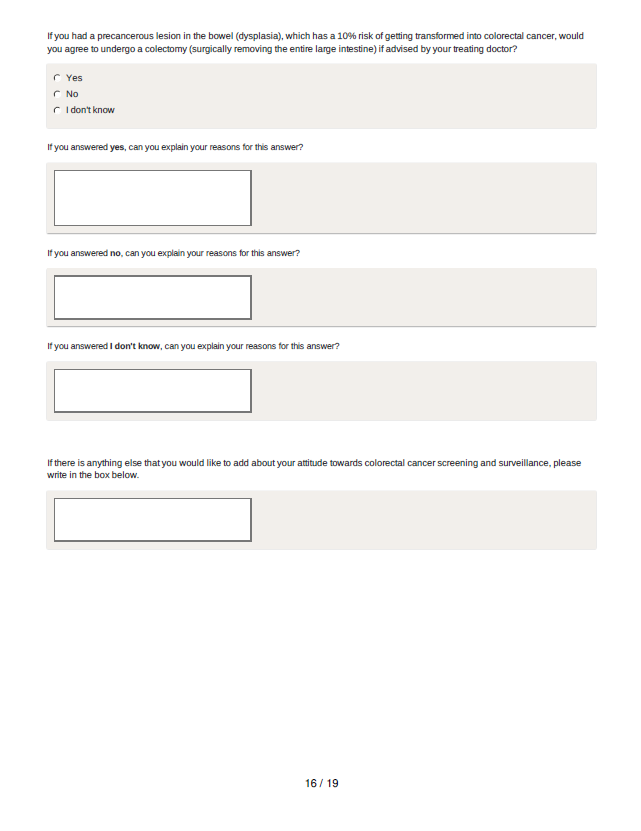
**

**
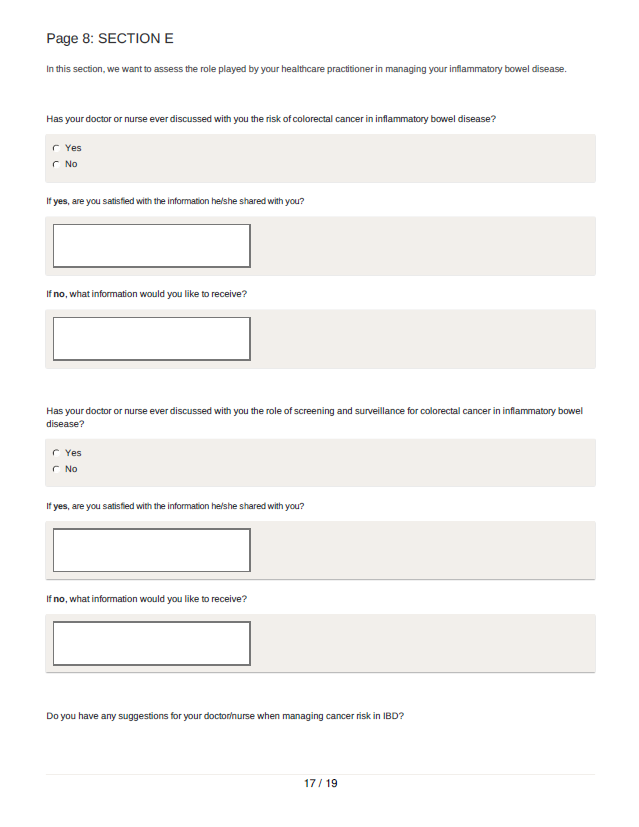
**

**
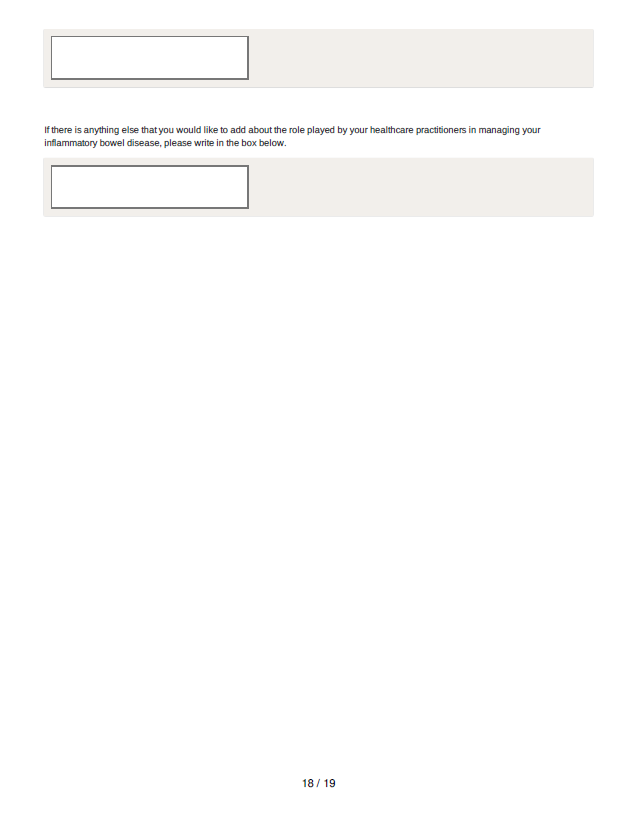
**

**
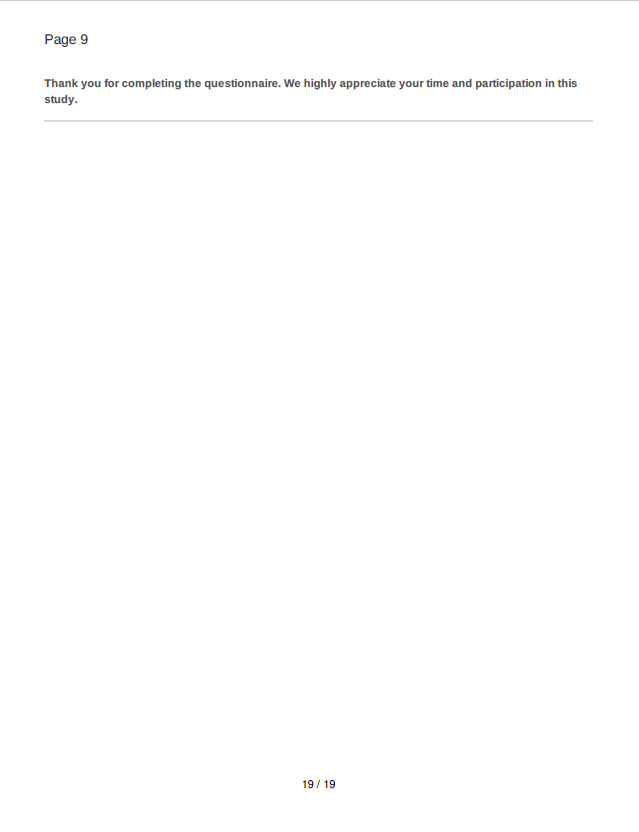
**
